# Diffuse large B‐cell lymphoma in a patient with dermatomyositis‐associated interstitial lung disease: A case report

**DOI:** 10.1111/1759-7714.13171

**Published:** 2019-08-26

**Authors:** Shan Li, Yuxin Sun, Chi Shao, Kai Xu, Hui Huang

**Affiliations:** ^1^ Department of Pulmonary and Critical Care Medicine Peking Union Medical College Hospital, Chinese Academy of Medical Sciences & Peking Union Medical College Beijing China; ^2^ Radiological Department Peking Union Medical College Hospital, Chinese Academy of Medical Sciences & Peking Union Medical College Beijing China

**Keywords:** Dermatomyositis, idiopathic inflammatory myositis, interstitial lung disease, lymphoma, malignancy

## Abstract

A 63‐year‐old man presented to our clinic complaining of cough and exertional dyspnea. He was diagnosed with dermatomyositis‐associated interstitial lung disease (DM‐ILD) with typical rashes, an elevated creatine kinase level and chest high‐resolution computed tomography (CT) manifestations. His symptoms and lung shadows improved after treatment with corticosteroids, immunosuppressants and pirfenidone, although his serum creatine kinase level remained elevated. An expanding nodule in the lower left lung and lymphadenopathy in the right cardiophrenic angle were visible on the repeated chest CT scan during follow‐up approximately one year later. Empirical antibiotics had no effect. A positron emission tomography‐computed tomography (PET‐CT) scan showed an increased standard uptake value (SUV) in the newly emerged pulmonary nodule, mediastinal lymphadenopathy and multiple hepatic masses. He was diagnosed with diffuse large B‐cell lymphoma following liver biopsy. After eight cycles of rituximab‐cyclophosphamide, doxorubicin, vincristine, and prednisone (R‐CHOP) chemotherapy, the lymphoma was cured clinically, and his interstitial lung disease (ILD) had improved. Tumor surveillance should be performed during DM‐ILD follow‐up, and rituximab could be a promising choice for DM‐ILD concurrent with lymphoma.

## Introduction

Malignancy and autoimmune diseases such as scleroderma and idiopathic inflammatory myositis (IIM) are associated with each other.^1^ Dermatomyositis (DM) is considered a clinical subtype of IIM with a higher risk of malignancy. The standardized incidence ratio (SIR) for IIM combined with malignancy ranges from 2.2 to 6.5. In a review of Asian patients with myositis and malignancy, nasopharyngeal and lung cancers were the most common tumors, and interstitial lung disease (ILD) was considered a protective factor for malignancy in IIM patients.^2^ Here, we report a DM‐ILD patient with diffuse large B‐cell lymphoma after treatment for one year with corticosteroids, immunosuppressants and pirfenidone. The lymphoma was clinically cured, and the ILD had improved following eight cycles of rituximab and chemotherapy.

## Case report

**Figure 1:**
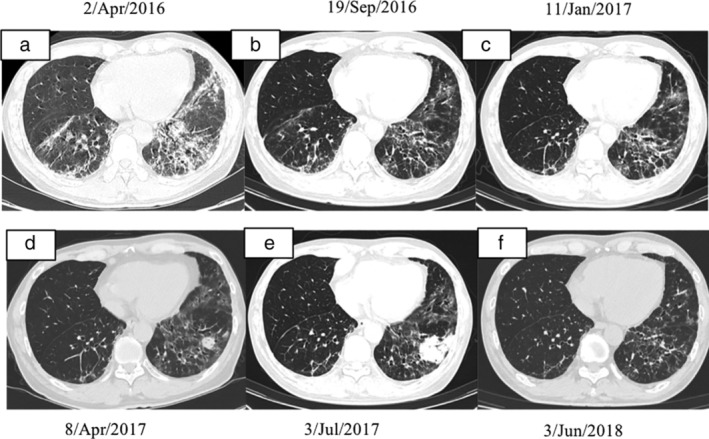
Serial high‐resolution CT scans of the chest. (**a**) The CT scan on 2 April 2016 showed reticular opacities, irregular linear opacities and diffuse ground‐glass opacities in both inferior lungs and left lingual lobe. (**b**) The CT scan on 19 September 2016 showed that ground glass opacities in both inferior lungs and left lingual lobe was improved after treatment. (**c**) The CT scan on 11 January 2017 showed that ground glass opacities in both inferior lungs and left lingual lobe was stable during follow‐up. (**d**) The CT scan on 8 April 2017 showed a new onset nodule in the left inferior lung, and the interstitial lung disease was stable. (**e**) The CT scan on 3 July 2017 showed the pulmonary nodule in the left inferior lung was obviously enlarged, but the ground glass opacities were stable. (**f**) The CT scan on 3 June 2018 showed that the left inferior pulmonary nodule had disappeared, and the ground‐glass opacities in both lungs had also improved.

A 63‐year‐old male patient with no previous medical history presented to our clinic complaining of a dry cough and exertional dyspnea on 26 April 2016. He also had myalgia and symmetrical proximal weakness, and could hardly lift his feet higher than 5 cm. He had no fever and no hemoptysis. He smoked half a pack daily for almost 20 years, but had quit smoking 10 years previously. He had classical rashes, including heliotrope rash and Gottron's sign, and Velcro rales could be heard in both basal lungs. His proximal muscle strength was approximately grade 4–5 in both upper and lower extremities. Complete blood count was approximately normal, but the blood biochemical panel showed an elevation in alanine transaminase (ALT; 196 U/L), aspartate transaminase (AST; 204 U/L), lactate dehydrogenase (LDH; 882 U/L) and creatine kinase (CK; 7751 U/L). Serum creatinine (Cr) and cardiac troponin I levels were normal. The erythrocyte sedimentation rate (ESR) was 49 mm/hour, and high‐sensitivity C‐reactive protein (hsCRP) was normal. The 18‐item antinuclear antibodies (ANA) panel showed a positive antinuclear antibody with a titer of 1:320 (cytoplasmic type) and a positive anti‐Ro52 antibody. The anti‐Ro52 antibody was the unique abnormal antibody in the 16‐item myositis antibody panel. A pulmonary function test (PFT) showed restrictive and diffusion impairment. Chest CT showed bilateral reticular opacities, irregular linear opacities, and ground‐glass patches in both inferior lungs (Fig 1a). The echocardiogram (ECG), abdominal and pelvic CT scans, and stool test was normal.

**Figure 2:**
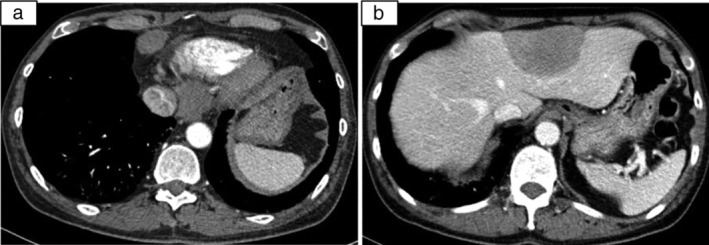
Contrast chest and abdominal CT showed enlarged mediastinal and retroperitoneal lymphadenopathy (**a**) and a huge left hepatic mass (**b**).

According to the criteria of Bohan and Peter 1975,^3^, ^4^ he was diagnosed with dermatomyositis‐associated interstitial lung disease (DM‐ILD). After two days of intravenous methylprednisolone (80 mg b.i.d), his CK decreased to 3541 U/L. The administration of oral prednisone (with an initial dosage of 1 mg/kg/day) and cyclophosphamide (CTX, approximately 2 mg/kg/day) were continued. The respiratory symptoms and muscle weakness had improved two weeks later, and prednisone was tapered gradually (minus 2.5 mg/week and stayed 15 mg/day). The serum CK decreased significantly one month later, and remained at approximately 500–750 U/L over the following period, although the patient did not report any weakness. Repeat chest CT in September 2016 (Fig 1b) showed improvement in the lung shadows. He was administered prednisone (15 mg/day), cyclophosphamide (CTX) and pirfenidone in the following three months. As his serum CK level and ILD had not further improved in January 2017 (Fig 1c), CTX was replaced with mycophenolate mofetil (MMF). In April 2017, he complained of cough but without any fever or sputum. Although the ILD imaging was almost the same as that obtained in January, there was a nodule in the left inferior lung (Fig 1d). He had no fever and no sputum. The serum *Legionella pneumophila* antibody, cryptococcal antigen and interferon‐γ release assays were all negative. Empirical treatment with moxifloxacin and azithromycin was subsequently prescribed. His cough was relieved, but the pulmonary nodule had obviously enlarged on the repeated HRCT scan in July 2017 (Fig 1e). The subsequent contrast CT scan and positron emission tomography‐computed tomography (PET‐CT) scan demonstrated an elevated standard uptake value (SUV), ranging from 12 to 30.1 for the pulmonary nodule, enlarged mediastinal lymphadenopathy and left hepatic mass (Fig. 2).

He was diagnosed with diffuse large B‐cell lymphoma (activated B‐cell subtype) after liver biopsy. After four cycles of chemotherapy with rituximab, CTX, doxorubicin, vincristine, and prednisone (R‐CHOP), the tumor disappeared in the repeated PET‐CT scan, and the serum CK level returned to normal. After another four cycles of R‐CHOP chemotherapy, the lymphoma seemed to be cured clinically from the repeated PET‐CT scan, and his ILD had improved (Fig 1f). Chemotherapy was ceased according to the advice of the hematologist. The patient took prednisone (7.5 mg once a day) for his ILD and pirfenidone (0.6 g t.i.d.) throughout his chemotherapy and continued for another six months. The prednisone was then tapered gradually and he stopped taking the prednisone and pirfenidone in November 2018. From then on, a serum biochemical panel, a chest and abdominal CT scan and a PFT were performed every three months. Both the ILD and the lymphoma remain stable.

## Discussion

Since the first report by Stertz in 1916, the association between inflammatory myositis and malignancy has been discussed extensively.^2^, ^5^, ^6^, ^7^, ^8^, ^9^, ^10^, ^11^, ^12^ Although there were differences among these studies, it has been well reported that both polymyositis (PM) and DM patients are at a higher risk of malignancy than non‐PM/DM patients. Most studies have shown that DM has greater association with malignancy than PM.^2^, ^9^, ^10^, ^11^, ^13^, ^14^

The age‐ and sex‐adjusted SIR of malignancy for DM patients was higher than that for PM patients. The risk factors for malignancy were reported as male sex, an age older than 45 years, the presence of skin ulcerations (especially skin necrosis), increased serum CK and inflammatory markers, positive anti‐transcriptional intermediary factor‐1γ (TIF‐1γ) autoantibodies and being within one year of the diagnosis of DM.^2^, ^9^, ^10^, ^13^ However, the meta‐analysis of Best *et al*. showed that positive anti‐TIF‐1γ was more common in solid organ cancers than in hematological malignancies.^12^ Other factors, including ILD, Raynaud's phenomenon and positive anti‐JO‐1 antibody, have been reported as protective factors for malignancy.^2^, ^13^

**Figure 3:**
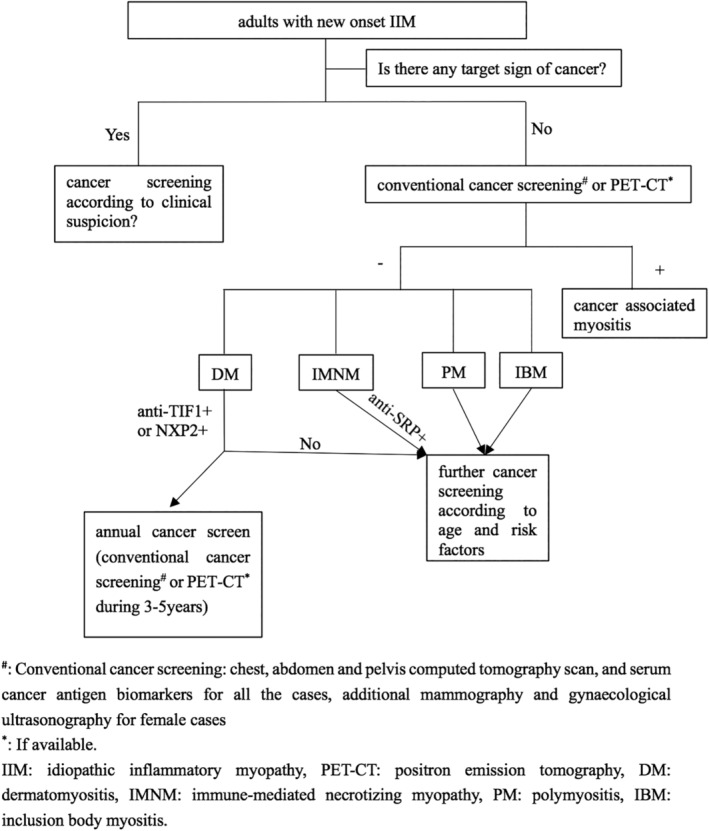
Suggested algorithm for cancer screening in adult patients with new onset idiopathic inflammatory myositis (IIM).

In our patient, the initial cancer screen including chest, abdominal and pelvic CT and stool test was negative, and he was diagnosed with DM‐ILD. However, he was a 63‐year‐old male, and his serum CK remained increased. We were aware of the concurrent risk of cancer. Because a new pulmonary nodule showed up approximately one year after the diagnosis of DM and became worse after administration of antibiotics, malignancy was suspected. A PET‐CT scan was performed because of the worsening lung shadow. With PET‐CT guidance, a liver biopsy confirmed the final diagnosis of lymphoma. Therefore, although there was no evidence‐based guideline for malignancy screening in PM/DM cases, malignancy screening, especially for a newly diagnosed myositis patient, was important. Some practical algorithms have been suggested for malignancy screening according to the presence or absence of predisposed cancer risk factors (Fig. 3).^2^, ^15^

The types of malignancy were not the same among different studies, and they varied with different regions and races.^2^, ^5^, ^11^ In the study by Marie *et al*., hematological malignancies, especially B‐cell lymphoma, were significantly associated with PM/DM.^16^ However, in the meta‐analysis of Ungprasert *et al*.^17^ lung and nasopharyngeal cancers were the most common malignancies in Asian populations. Our patient was diagnosed with large B‐cell lymphoma after liver biopsy. Rituximab has recently been recommended for refractory myositis cases.^18^ For our patient, with the diagnosis of diffuse large B‐cell lymphoma, R‐CHOP was effective for both the lymphoma and the DM‐ILD, and he responded well to chemotherapy.

The results of this study suggest that malignancy screening should be arranged for inflammatory myositis patients, especially within the first year for DM patients. Chemotherapy with rituximab is recommended for DM‐ILD patients with concurrent B‐cell lymphoma.

## Disclosure

No authors report any conflict of interest.
